# Relaxin reverses inflammatory and immune signals in aged hearts

**DOI:** 10.1371/journal.pone.0190935

**Published:** 2018-01-18

**Authors:** Brian Martin, Beth Ann Gabris-Weber, Rajiv Reddy, Guillermo Romero, Ansuman Chattopadhyay, Guy Salama

**Affiliations:** 1 Department of Bioengineering, University of Pittsburgh, Pittsburgh, PA, United States of America; 2 School of Medicine, Heart and Vascular Institute, University of Pittsburgh, Pittsburgh, PA, United States of America; 3 Department of Pharmacology & Chemical Biology, University of Pittsburgh, Pittsburgh, PA, United States of America; 4 Molecular Biology Information Service, Health Sciences Library System, University of Pittsburgh, Pittsburgh, PA, United States of America; Centro Cardiologico Monzino, ITALY

## Abstract

**Background:**

‘Healthy’ aging drives structural and functional changes in the heart including maladaptive electrical remodeling, fibrosis and inflammation, which lower the threshold for cardiovascular diseases such as heart failure (HF) and atrial fibrillation (AF). Despite mixed results in recent clinical trials, Relaxin-therapy for 2-days could reduce mortality by 37% at 180-days post-treatment, in patients with acute decompensated HF. Relaxin’s short life-span (hours) but long-lasting protective actions led us to test the hypothesis that relaxin acts at a genomic level to reverse maladaptive remodeling in aging and HF.

**Methods and results:**

Young (9-month) and aged (24-month), male and female F-344/Brown Norway rats were treated with relaxin (0.4 mg/kg/day) for 2-weeks delivered by subcutaneous osmotic mini-pumps or with sodium acetate (controls). The genomic effects of aging and relaxin were evaluated by extracting RNA from the left ventricles and analyzing genomic changes by RNA-sequencing, Ingenuity Pathway Analysis, MetaCore and tissue immunohistochemistry. We found that aging promotes a native inflammatory response with distinct sex-differences and relaxin suppresses transcription of multiple genes and signaling pathways associated with inflammation and HF in both genders. In addition, aging significantly increased: macrophage infiltration and atrial natriuretic peptide levels in female ventricles, and activation of the complement cascade, whereas relaxin reversed these age-related effects.

**Conclusion:**

These data support the hypothesis that relaxin alters gene transcription and suppresses inflammatory pathways and genes associated with HF and aging. Relaxin’s suppression of inflammation and fibrosis supports its potential as a therapy for cardiovascular and inflammation-related diseases, such as HF, AF and diabetes.

## Introduction

Aging is a major risk factor for atrial fibrillation (AF) [1] and heart failure (HF), [2] which may reach pandemic proportions due to an increasingly aging population, estimated to reach 70 million people over the age of 65 in the United States by 2030.[3] HF is defined as the inability of the heart muscle to pump enough blood to meet the energy demands of the body [4], and aging is associated with multiple maladaptive vascular and cardiac structural, electrical and functional changes that increase the susceptibly to HF, AF and other cardiovascular diseases (CDs). [3, 5] Structural remodeling in aging includes: increases in vascular stiffness, left ventricular (LV) and aortic wall thickness [3], fibrosis [6] and myocyte hypertrophy that promote diastolic dysfunction, coronary heart disease, [3] AF [7] and HF. [6] In sum, age-related maladaptive responses lower the threshold for CD development. [6] Recent evidence posits that aging brings-on the gradual development of a chronic, low-grade inflammation, termed “inflammaging” which is possibly the greatest risk factor for many age-related diseases. [8] Chronic inflammation can result from failed resolution of an injury by the body or stimuli resulting in asymptomatic responses.[9] Inflammation has been linked to multiple CDs including myocardial infarction, resulting in increased leukocyte infiltration that leads to pathological left ventricular (LV) remodeling.[10] In addition, complement cascade activity can be activated in the presence of pathogens or tissue injury and has been linked to higher hospitalizations and mortality in ischemia/reperfusion studies, [9, 11] and to inflammation associated with insulin resistance and type II diabetes. [12] Interleukin (IL)-1 and IL-6 have been linked to cardiac hypertrophy, reduced contractility, increased arrhythmia susceptibility, increased C-reactive protein, coronary artery disease [13] and raise stroke risk, while tumor necrosis factor alpha (TNFα) is associated with LV dilatation and dysfunction and HF progression. [14–16] In addition to inflammatory markers of disease, multiple markers are used clinically to determine HF progression and prognosis. Brain natriuretic peptide (BNP, *gene*: *NPPB*) is a marker of HF, is indicative of hemodynamic wall stress and is a risk factor for AF and HF. [15, 17, 18] Like BNP, atrial natriuretic peptide (ANP, *gene*: *NPPA*) is a marker of congestive HF [19] and growth differentiation factor (GDF)-15 is implicated in HF and inflammatory and oxidative stress. [18, 20] Clinical trials that suppressed the inflammatory-related cytokine, TNFα, have yielded disappointing results in chronic HF patients. TNFα chelation had no effect on primary endpoints of hospitalization and death, leading investigators to hypothesize a large degree of redundancy in the immune response related to HF, so that targeting a single cytokine may prove to be ineffective. [11] Studies in patients with rheumatoid arthritis using tocilizumab to block IL-6 signaling through the IL-6 receptor have shown beneficial effects, though resulted in side-effects including pro-atherogenic dyslipidemia and increased low density lipoprotein (LDL), [13] which is associated with increased risk of coronary artery disease. [21]

Although age is a major risk factor for inflammation, AF and HF, [8, 22, 23] with few exceptions [23–25] there is a noticeable paucity of genetic studies on how global gene expression is altered in normal, ‘healthy’ cardiac aging. Many were done in mice despite the challenge of comparing age-related gene expression patterns in mice and man. Yet, differences in mice may be due to a non-uniform rate of aging in mice.[24] since other studies identified aging gene expression signatures that are conserved in mice, rats and humans, namely, inflammation and the immune response. [23]. This suggests that genomic studies of inflammation in rodents may be relevant to the development of therapies for disorders related to age and inflammation. Furthermore, the challenges of using mouse models of aging are largely dispelled through the use of the F-344 Brown/Norway rat model from the National Institute of Aging, which has been developed and used extensively to study the effects of aging due to its homogeneous and linear rate of aging, increased cardiac collagen production and increased susceptibility to atrial fibrillation and systolic and diastolic dysfunction; all hallmarks of human aging. [7, 26, 27]

Relaxin (RLX), a hormone of pregnancy, has shown significant cardiovascular benefits including a significant reduction in AF susceptibility in aged [5] and hypertensive [28] rats, primarily through a reversal of fibrosis and an increase in voltage gated sodium channels, Nav1.5, and its current, I_Na_. In phase II clinical trials, RLX reduced 180-day cardiovascular and all-cause mortality in acute decompensated HF patients after 48-hour RLX infusion; [29, 30] however, recent Phase III clinical trials failed to meet the primary endpoint of reduced mortality in HF patients (https://www.novartis.com/ news release, March 2017), though details are yet to be published. In spite of these results, RLX has been shown to be a potent anti-fibrotic through regulation of fibroblast activation, collagen secretion and reductions in pro-fibrotic transcripts,[5, 31] a suppressor of AF through a combination of structural and electrical cardiac remodeling,[5, 28] and to increase vascular compliance and reduce systemic vascular resistance.[31, 32] In addition, we report here RLX’s anti-inflammatory actions, suggesting that RLX acts on multiple signaling pathways and may be a therapy for various diseases associated with these pathological conditions. Despite growing evidence of RLX’s beneficial effects,[5, 28, 33–35] the mechanisms underlying these effects remain unclear. In RELAX-AHF trials, RLX serum concentrations rose rapidly, levelled off after 4 hours of infusion, and decreased quickly post-infusion.[36] The short lifespan of RLX (~2hrs) suggests that the long-term benefits of RLX stem from long-lasting genomic effects rather than an improvement of hemodynamics at the time of infusion.[37] Here, we test the hypothesis that RLX acts at the genomic level to modify HF and AF related genes and signaling pathways that are activated or inhibited in ‘healthy/normal’ aging. We show that aging significantly increases gene transcription of multiple pro-inflammatory and HF related genes and signaling pathways. Significantly, we show that RLX reverses the effects of aging in both genders through genetic regulation of these pathways and that aging increases macrophage accumulation in females, but not male left ventricles, which is reversed by RLX treatment. These data provide evidence in support of RLX as a therapy for a plethora of inflammatory diseases through its regulation of multiple cytokines and signaling pathways.

## Materials and methods

### Animal model

Young (9-month-old) and aged (24-month-old) male and female F-344/Brown Norway rats were obtained from the National Institute of Aging. Rats were treated with vehicle (sodium acetate) or RLX (400 μg/kg/day) for 14 days via subcutaneous osmotic mini pumps. Left ventricular tissue was snap frozen in liquid nitrogen and stored at -80 degrees Celsius until use. Studies were performed in accordance with the Guide for the Care and Use of Laboratory Animals and were approved by the Institutional Animal Care and Use Committee at the University of Pittsburgh.

### RNA-sequencing

Three animals were analyzed from male groups and four animals from female groups (24-month-old ± RLX, 9-month-old ± RLX). A total of 1 μg of RNA was sent to the University of Pittsburgh Genomics Core for sequencing and library preparation. Library preparation was performed using the TruSEQ Stranded Total RNA Sample Preparation Kit (Illumina, San Diego, CA) according to manufacturer’s instructions. Following removal of ribosomal sequences, RNA was fragmented for 8 minutes and reverse transcription performed. Double stranded cDNA was subjected to 3’ adenylation and ligation of sequencing adapters. Sequencing was carried out on a NextSeq 500 (Illumina) for 2 x 75 bp paired end reads. Loading concentration was 1.4 pM for females and 1.2 pM for males.

### RNA-sequencing analysis

Raw transcript data was imported into Biomedical Genomics Workbench 3.0.1 and reads were mapped to the rat reference genome. Differentially expressed genes (DEGs) used in pathway analysis were determined between old and young rats with and without RLX using filters to select genes with Reads Per Kilobase of transcript per Million, RPKM ≥ 1, absolute fold change (FC) > 1.5 using total gene count and a *false discovery rate* (FDR) ≤ 0.05. Fisher’s exact test was used to test for pathway significance. Activation (z-score > 2, orange bars) or inhibition (z-score < 2, blue bars) of each pathway was determined by *Ingenuity Pathway Analysis* (IPA) and is a measure of experimentally determined gene expression changes reported in the literature. For reporting of individual genes, data are presented as FC with p-value and FDR. DEGs filtered for pathway analysis were imported into Ingenuity Pathway Analysis and MetaCore to determine signaling pathways and upstream regulators affected by aging or RLX treatment. Aging studies were carried out by comparing RNA-seq data from 24-month old untreated (UNT) vs. 9-month old UNT rats. RLXs effect was measured by comparing 24-month-old + RLX vs. 24-month-old UNT rats. The data from this publication have been deposited in NCBI’s Gene Expression Omnibus (GEO), and are accessible via GEO series number GSE106377 at https://www.ncbi.nlm.nih.gov/geo/query/acc.cgi?acc=GSE106377.

### Immunofluorescence

Male (n = 3-4/group) and Female (n = 5/group) left ventricular tissue were thawed and fixed in 2% PFA at room temperature and left in 30% sucrose for 24 hours at 4 degrees Celsius. LV tissue sections (7 μm) were placed in 0.1% Triton 100-x for 10 minutes. Sections were blocked with 2% BSA for 30 minutes followed by a 90-minute incubation with primary antibodies, rabbit-anti-F4/80 polyclonal (Thermo Fischer, PA5-21399), mouse-anti-CD4 monoclonal (BioRad, MCA55GA), and mouse-anti ANP monoclonal (Santa Cruz Biotechnology, sc-515701) antibodies. Sections were washed 3x 5minutes and incubated with secondary antibodies for 45 min and another wash. Finally, sections were stained for 1 minute with DAPI and coverslips mounted with gelvatol. Images were taken on an Olympus Fluoview inverted confocal microscope at 60x magnification. Image parameters were obtained for one image and kept constant throughout imaging. Images were analyzed using ImageJ software and only circular staining with a clear association with a nucleus were included in the macrophage and T-cell analysis. ANOVA was used to determine statistically significant differences between groups with Tukey’s post hoc analysis. Data is presented as mean ± SEM. P<0.05 was considered statistically significant.

## Results

### Effects of aging and relaxin on biomarkers of AF and HF

In the clinical setting, several markers have been associated with the severity and/or mortality of AF and HF patients. In a proteomics study of AF, Lind et al [15] reported an association of multiple proteins with AF using two cohorts. The primary cohort included both genders and a validation cohort included only males. It is interesting to note that they reported thirteen AF associated proteins in the mixed gender cohort, but merely 5 in the male only cohort, suggesting marked gender differences. Table 1 lists age and RLX-dependent transcription changes of multiple markers thought to be involved in AF and HF development. [14–18, 38–41]

**Table 1:** CD related genes.

RNA-seq analysis of cardiovascular disease related genes				
	Female Fold Change (p-value, FDR)		Male Fold Change (p-value, FDR)	
Gene	Aging	RLX	Aging	RLX
**IL-1β**	-1.24 (ns)	1.48 (ns)	-1.69 (0.02, ns)	-3.54 (<0.0001, <0.01) ✓✓
**IL-6**	1.82 (0.03, ns) ✓	-1.81 (0.04, ns) ✓	1.15 (ns)	-4.35 (<0.0001, 0.01) ✓✓
IL-6RA	1.09 (ns)	-1.20 (ns)	-1.10 (ns)	1.21 (ns)
CRP	Undetected	Undetected	Undetected	Undetected
TNFα	-1.63 (ns) ✓	2.67 (<0.01, ns) ✓	-1.87 (<0.01, ns) ✓	-1.82 (0.03, ns) ✓
**NPPB**	1.00 (ns)	1.28 (ns)	1.43 (0.04, ns) ✓	-1.84 (<0.0001, 0.01) ✓✓
**NPPA**	10.48 (<0.0001, <0.0001) ✓✓	-2.95 (0.01, ns) ✓	1.37 (ns)	-1.36 (ns)
**GDF-15**	-1.45 (0.07, ns)	1.37 (ns)	1.40 (ns)	-3.58 (<0.0001, 0.03) ✓✓
SPP1	2.64 (<0.01, ns) ✓	-2.24 (<0.01, ns) ✓	-1.10 (ns)	-3.42 (<0.01, ns) ✓
CST3	1.35 (0.01, ns)	-1.47 (0.04, ns)	1.02 (ns)	-1.46 (<0.01, ns)
**IFNG**	6.10 (<0.0001, <0.01) ✓✓	-3.25 (<0.01, ns) ✓	-2.48 (0.03, ns) ✓	1.09 (ns)
FGF-23	1.41 (ns)	-1.14 (ns)	-4.44 (<0.01, ns) ✓	1.28 (ns)
FABP4	1.25 (ns)	-1.09 (ns)	1.81 (<0.01, ns) ✓	-1.03 (ns)
CSTB	1.28 (0.02, ns)	-1.18 (ns)	1.39 (ns)	-1.47 (ns)
IL-1RA	1.78 (<0.01, ns) ✓	-1.32 (ns)	2.11 (0.04, ns) ✓	-2.12 (0.03, ns) ✓
TNF-R1	-1.34 (0.04, ns)	1.14 (ns)	-1.16 (ns)	-1.16 (ns)
TNF-R2	1.05 (ns)	-1.17 (ns)	-1.19 (ns)	1.18 (ns)
Plaur_1	1.2 (ns)	-1.6 (0.02, ns) ✓	-1.21 (ns)	-1.39 (ns)
Plaur_2	1.77 (ns)	-1.76 (ns)	1.85 (ns)	-5.56 (<0.01, ns) ✓
Plaur_3	2.65 (0.01, ns) ✓	-3.55 (<0.01, ns) ✓	-1.2 (ns)	-5.29 (0.01, ns) ✓
CSF-1	1.4 (<0.01, ns)	-1.18 (ns)	1.23 (ns)	1.21 (ns)
AM	1.42 (0.01, ns)	-1.25 (ns)	1.06 (ns)	-1.14 (ns)
TNNT2_1	-1.05 (ns)	1.21 (ns)	1.3 (ns)	-1.53 (<0.01, ns) ✓
TNNT2_2	-1.13 (ns)	1.2 (ns)	1.26 (ns)	-1.26 (ns)
**LDLR**	-1.91 (<0.01, 0.05) ✓✓	1.56 (0.01, ns) ✓	-2.52 (<0.01, ns) ✓	1.32 (ns)

FDR: False Discovery Rate. Multiple test correction technique used in RNA-sequencing data analysis. Underscores indicated gene isoform. **Bold**: at least one group shows FDR < 0.05. ns: not significant. Yellow,✓✓: FDR<0.05. Blue,✓: Fold Change > 1.5, p<0.05.

RNA-sequencing (RNA-seq) analysis showed that aging and aging plus RLX-treatment altered with high significance (FDR<0.05) more than 7 genes associated with HF and AF in the clinical setting. In Table 1, these genes are highlighted in bold and dark shading and genes exhibiting highly significant changes have double-check marks. In females, aging or RLX altered atrial natriuretic peptide (NPPA), interferon gamma (INFG) and the LDL receptor (LDLR). In males, aging or RLX altered IL-1β, IL-6, NPPB, and GDF-15. In addition, several genes exhibited > 1.5 absolute fold changes with p<0.05, but do not reach the more stringent criterion of FDR<0.05 and are labeled with a single check mark. RLX significantly *reversed* multiple transcripts associated with HF and AF and therefore appears to not only stop the progression but to *reverses* the pathological expression of many cytokines.

**Fig 1:**
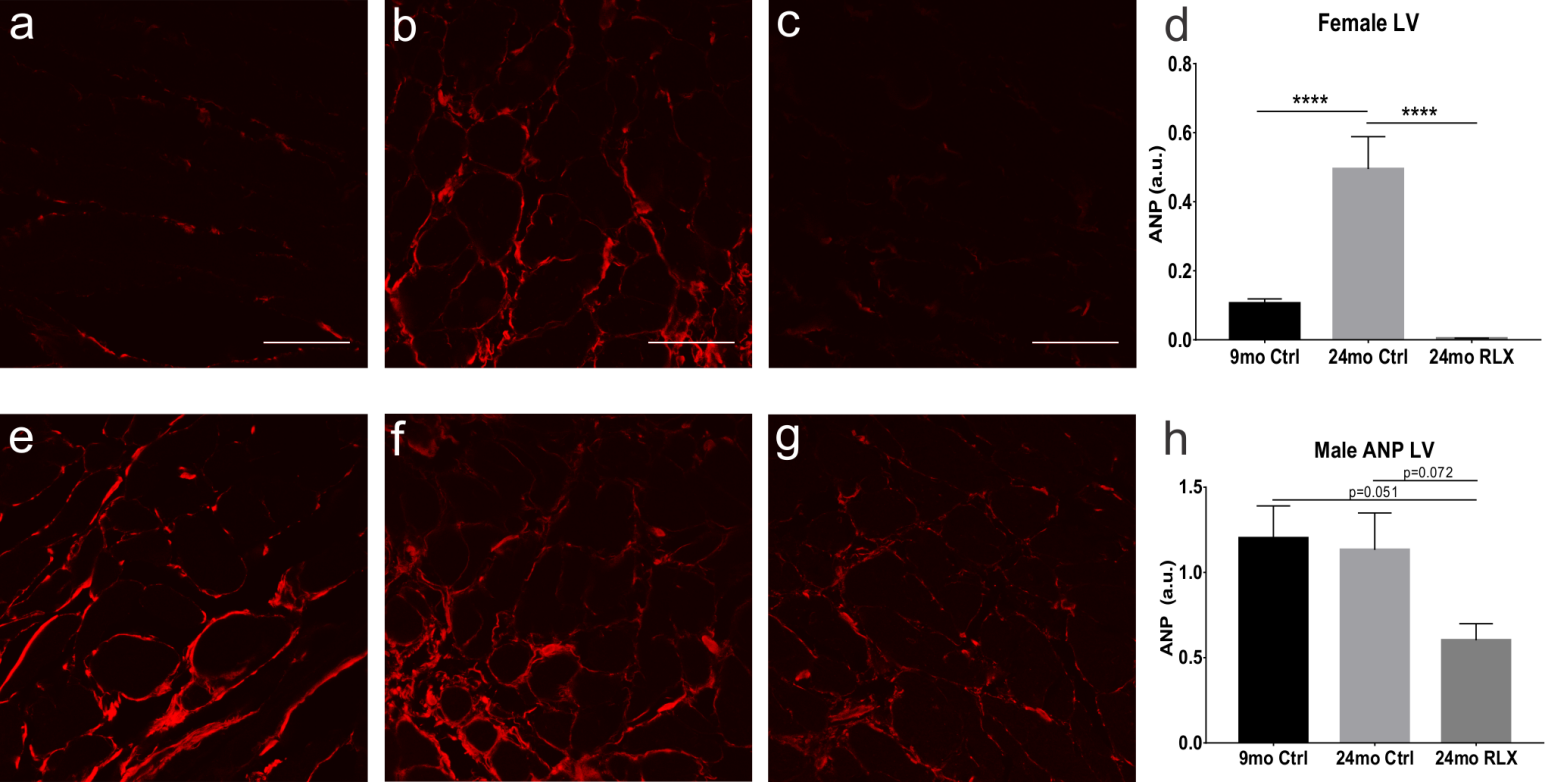
Protein expression of ANP in female and male heart. **A-D. Female.** Female aging (B) resulted in upregulation of ANP expression compared to young controls (A) and was reversed by RLX (C). D. Quantification of ANP expression in female LV. **E-H. Male.** In male rats, aging (F) did not increase ANP expression compared to young controls (E), though there was a reduction in ANP with RLX treatment (G) though this did not reach significance compared to young or aged males. H. Quantification of ANP expression in male LV. Red: ANP. ****: p<0.0001. Scale bar: 50μm.

Atrial natriuretic peptide (ANP), the most differentially expressed gene in female rats, was selected for further protein expression analysis to confirm that the effects of RLX on gene expression had functional consequences. In female rats, 9-month-old controls (Fig 1A) exhibited significantly less ANP than in aged ventricles (Fig 1B). RLX treatment (Fig 1C) significantly reversed aging’s effect on ANP expression. In male rats, there was no significant change in ANP expression in the aged versus the young controls (Fig 1E, 1F and 1H), though there was a reduction in ANP with RLX treatment (Fig 1G and 1H), that did not reach statistical significance. These data match closely the gene expression data in Table 1 (NPPA) for ANP expression.

Upstream analysis (Table 2) searches for differentially expressed genes (DEGs) to predict activation or inhibition of transcription regulators and to determine which molecules can explain gene expression alterations observed in the data sets. Observed DEGs are compared to a database of known interactions with transcriptional regulators kept by Ingenuity, with transcripts grouped together under common regulators. The quantity of genes controlled by a given transcriptional regulator and the agreement of direction (+/-) of fold change of each DEG to that reported in the literature, leads to a prediction of activation or inhibition of those transcriptional regulators.

**Table 2:** Ingenuity pathway analysis: Upstream analysis.

Upstream analysis of regulators				
	Female		Male	
Upstream regulator	Aging	RLX	Aging	RLX
Hydrogen Peroxide		Inhibited		Inhibited
NFkB (complex)	Activated	Inhibited		Inhibited
TLR4	Activated			Inhibited
IFNG	Activated	Inhibited		Inhibited
IL6	Activated	Inhibited		Inhibited
LPS	Activated	Inhibited		Inhibited
Sirolimus	Inhibited			Activated
TGFβ1	Activated	Inhibited		Inhibited
SMAD7	Inhibited	Activated	Activated	

Consistent with the effects of aging shown in Table 1, NFkB signaling, a crucial pathway regulating many inflammatory genes, is upregulated in female aging and inhibited by RLX in both female and male rats. In female rats, IFNγ, IL-6, lipopolysaccharide (LPS) and transforming growth factor (TGF) β1 are activated in aging and inhibited by RLX. Interestingly, SMAD7, an inhibitor of TGFβ1 and fibrosis, is inhibited with aging and activated by RLX; consistent with the anti-fibrosis properties of RLX. In aged *male* rats, upstream analysis failed to detect an activation of the genes that were activated in aged female ventricles, yet RLX still inhibited many of them in aged male ventricles. Note that RLX inhibited several upstream regulators: hydrogen peroxide, NFkB, TLR4, IFNG, IL-6 and LPS in males and females (Table 2).

Besides classic inflammatory gene expression that change in HF, and aging ± RLX, RLX altered chemo-attractant genes involved in recruiting inflammatory and immune cells to sites of injury (Table 3).

**Table 3:** Chemokine and chemokine receptor transcripts.

Chemokine gene expression analysis				
	Female Fold change (p-value, FDR)		Male Fold change (p-value, FDR)	
Gene	Aging	RLX	Aging	RLX
**CCL1**	-1.02 (ns)	1.46 (ns)	-3.64 (<0.0001, 0.03) ✓✓	-1.21 (ns)
**CCL2**	1.23 (ns)	1.23 (ns)	1.43 (ns)	-3.26 (<0.0001, 0.04) ✓✓
CCL3	1.23 (ns)	-2.62 (<0.01, ns) ✓	-1.15 (ns)	1.89 (0.02, ns) ✓
**CCL5**	1.79 (<0.0001, <0.001) ✓✓	-1.54 (<0.01, ns)	-1.49 (<0.05, ns)	1.24 (ns)
**CCL6**	1.61 (<0.0001, <0.05) ✓✓	-1.27 (0.02, ns)	-1.56 (0.05, ns)	1.11 (ns)
**CCL19**	2.35 (<0.001, 0.01) ✓✓	-1.87 (<0.01, ns) ✓	-1.34 (ns)	-2.77 (<0.01, ns) ✓
CCL20	1.67 (ns)	-1.35 (ns)	3.07 (<0.01, ns) ✓	-1.37 (ns)
**CCL21**	-1.02 (ns)	-1.48 (<0.01, ns)	-2.55 (<0.0001, 0.01) ✓✓	-1.26 (ns)
**CXCL9**	1.55 (<0.01, 0.02) ✓✓	-1.67 (<0.01, 0.03) ✓✓	-1.10 (ns)	-1.09 (ns)
**CXCL13**	2.24 (<0.0001, <0.0001) ✓✓	-1.52 (<0.01, ns)	-1.07 (ns)	1.29 (ns)
**CXCL16**	1.60 (<0.0001, <0.001) ✓✓	-1.27 (0.03, ns)	1.22 (ns)	-1.14 (ns)
CCR1	1.90 (<0.01, ns) ✓	1.00 (ns)	-2.01 (<0.01, ns) ✓	1.13 (ns)
CCR5	1.30 (ns)	-1.17 (ns)	-1.34 (ns)	1.18 (ns)

FDR: False Discovery Rate. Multiple test correction technique used in RNA-sequencing data analysis. **Bold**: at least one group shows FDR < 0.05. NS: not significant. Yellow,✓✓: FDR<0.05. Blue,✓: p<0.05.

### Signaling pathway analysis

**Fig 2:**
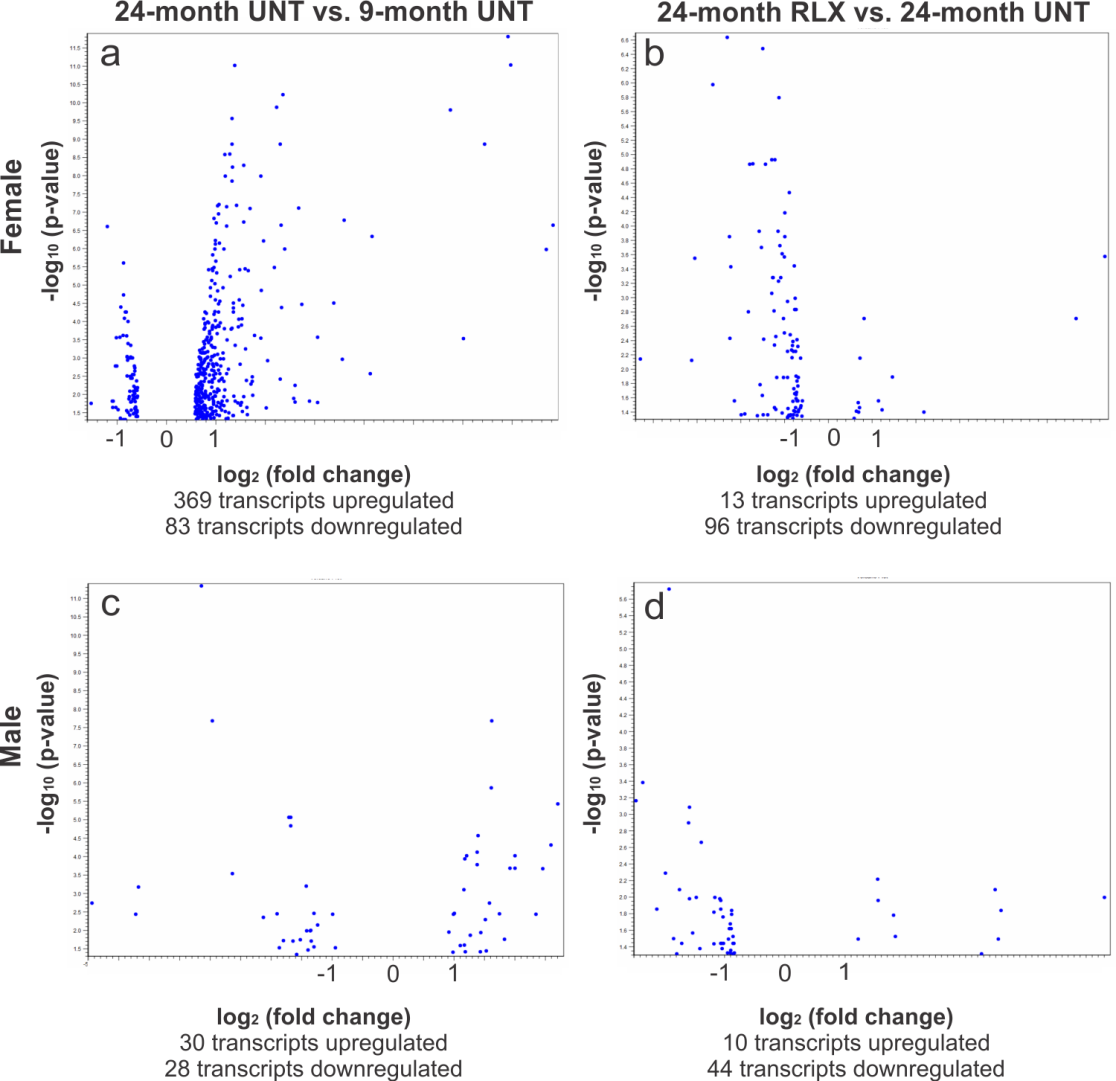
Volcano plots of differentially expressed genes in aging ± RLX treatment. A. Female aging resulted in an overall upregulation of transcripts, with fewer transcripts being downregulated. B. RLX treatment in females caused a marked suppression of most transcripts with few being upregulated. C. Transcript expression changes in males were muted compared to females during aging, with an even number of transcripts being up and downregulated. D. RLX treatment in males reflects that seen in females, with a substantial proportion of transcript alterations being suppressed compared to upregulated. Blue dots represent individual transcripts.

To identify signaling pathways that are affected by aging and RLX treatment, differentially expressed genes (DEGs) were filtered as transcripts whose absolute expression was altered by at least 1.5-fold, had expression values with RPKM (reads per kilobase of transcript per million) ≥ 1, and had a false discovery rate (FDR) < 0.05 (Fig 2).

### Female rats

**Fig 3:**
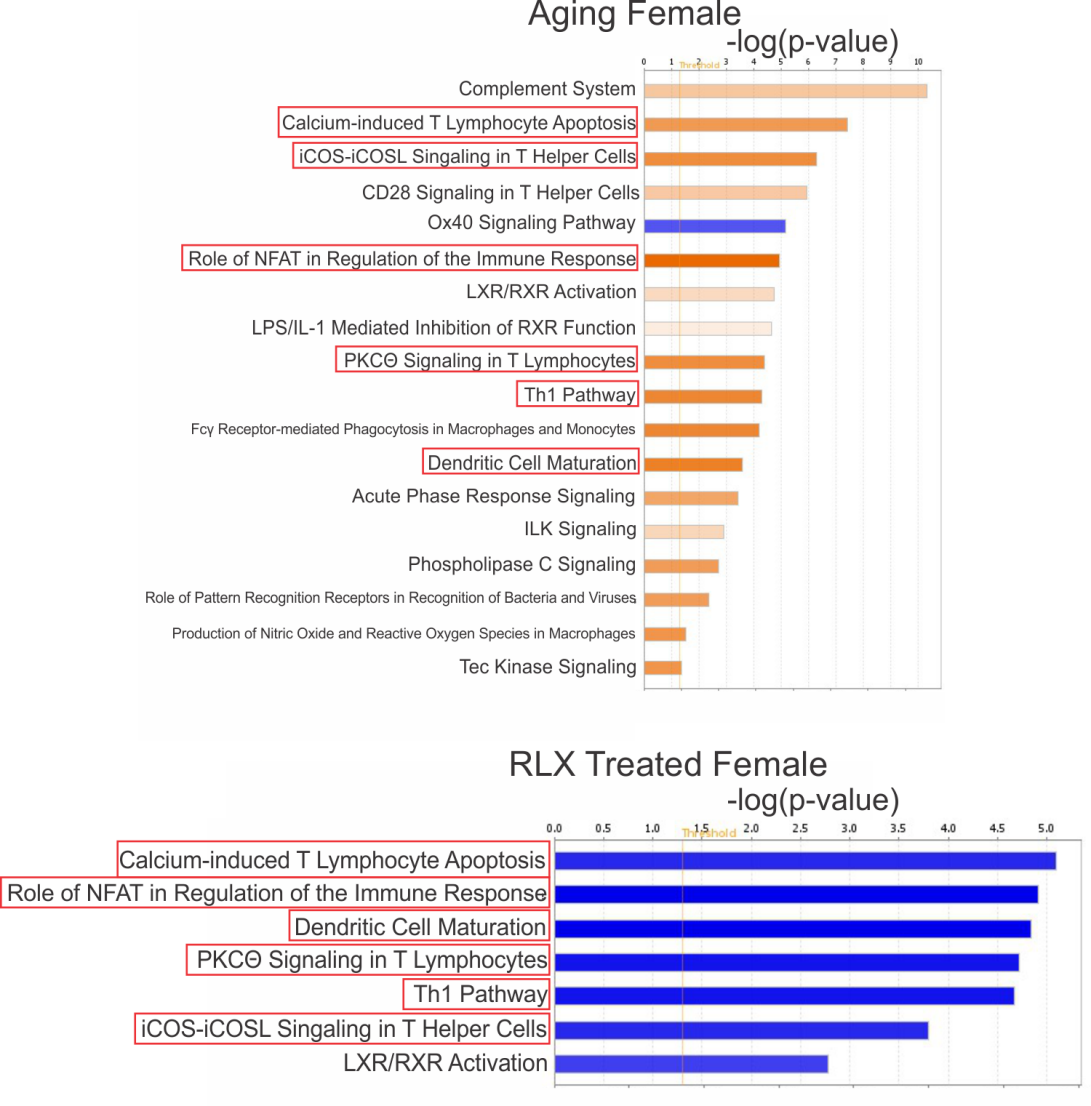
RLX inhibits multiple inflammatory pathways activated in aged females. (a) Pathway analysis of female rats suggest that multiple inflammatory signaling pathways are activated (orange) in aging and (b) RLX treatment inhibited (blue) many of these signaling pathways. Due to the substantial number of pathways altered in female aging (93 pathways) and RLX treatment (58 pathways), only those pathways whose activation or inhibition was predicted by IPA (orange or blue colors, respectively) are shown.

Application of Ingenuity Pathway Analysis (IPA) software to DEGs showed that female rats exhibit significant activation of inflammatory and immune signaling pathways with aging (Fig 3A) which are reversed by RLX treatment (Fig 3B). These pathways include: 1) Calcium-induced T lymphocyte apoptosis pathway, which acts to counter excessive inflammatory response [42, 43], though may be harmful in some cases [44], 2) PKCθ signaling in T Lymphocytes, involved in T-cell activation and NF-κB signaling [45, 46], 3) iCOS-iCOSL signaling in T-helper cells, resulting in activation and migration of lymphocytes and secretion of both anti- and pro-inflammatory cytokines [47], 4) NFAT regulation of the immune response, involved in cytokine production in leukocytes [48], 5) TH1 signaling, resulting in pro-inflammatory response, compared to the anti-inflammatory TH2 signaling [49] and 6) dendritic cell maturation, which acts as a link between innate and adaptive immunity [48].

Aging increased and RLX decreased expression of a common set of genes, namely those associated with the major histocompatibility complex (MHC) which is involved in immune system signaling (Table 4). This was supported by MetaCore (Thomson Reuters) analysis that showed activation of antigen presentation by MHC class I & II in aging and inhibition with RLX treatment (data not shown).

**Table 4:** Common genes altered by relaxin.

Gene reversal by relaxin in female rats			
Signaling pathway	Aging	RLX	RLXs Effect
Role of NFAT in Regulation of the Immune Response	Activates	Inhibits	FCGR3A/FCGR3B(↓), HLA-DQA1(↓), HLA-DRA(↓), HLA-DRB5(↓), LOC1009009593/RT(↓)
Dendritic Cell Maturation	Activates	Inhibits	
Calcium-induced T Lymphocyte Apoptosis	Activates	Inhibits	HLA-DQA1(↓), HLA-DRA(↓), HLA-DRB5(↓), LOC1009009593/RT(↓)
iCOS-iCOSL Signaling in T Helper Cells	Activates	Inhibits	
TH1 Pathway	Activates	Inhibits	
PKCθ Signaling in T Lymphocytes	Activates	Inhibits	

Right column indicates which genes are common between aging and RLX groups which RLX reverses compared to the aging group. Arrows indicated direction of change by RLX. FCGR3A/B: Encodes receptor for Fc portion of immunoglobin G. HLA: human leukocyte antigen; gene complex which forms the major histocompatibility complex. LOC: indicates genes with uncertain functions.

**Fig 4:**
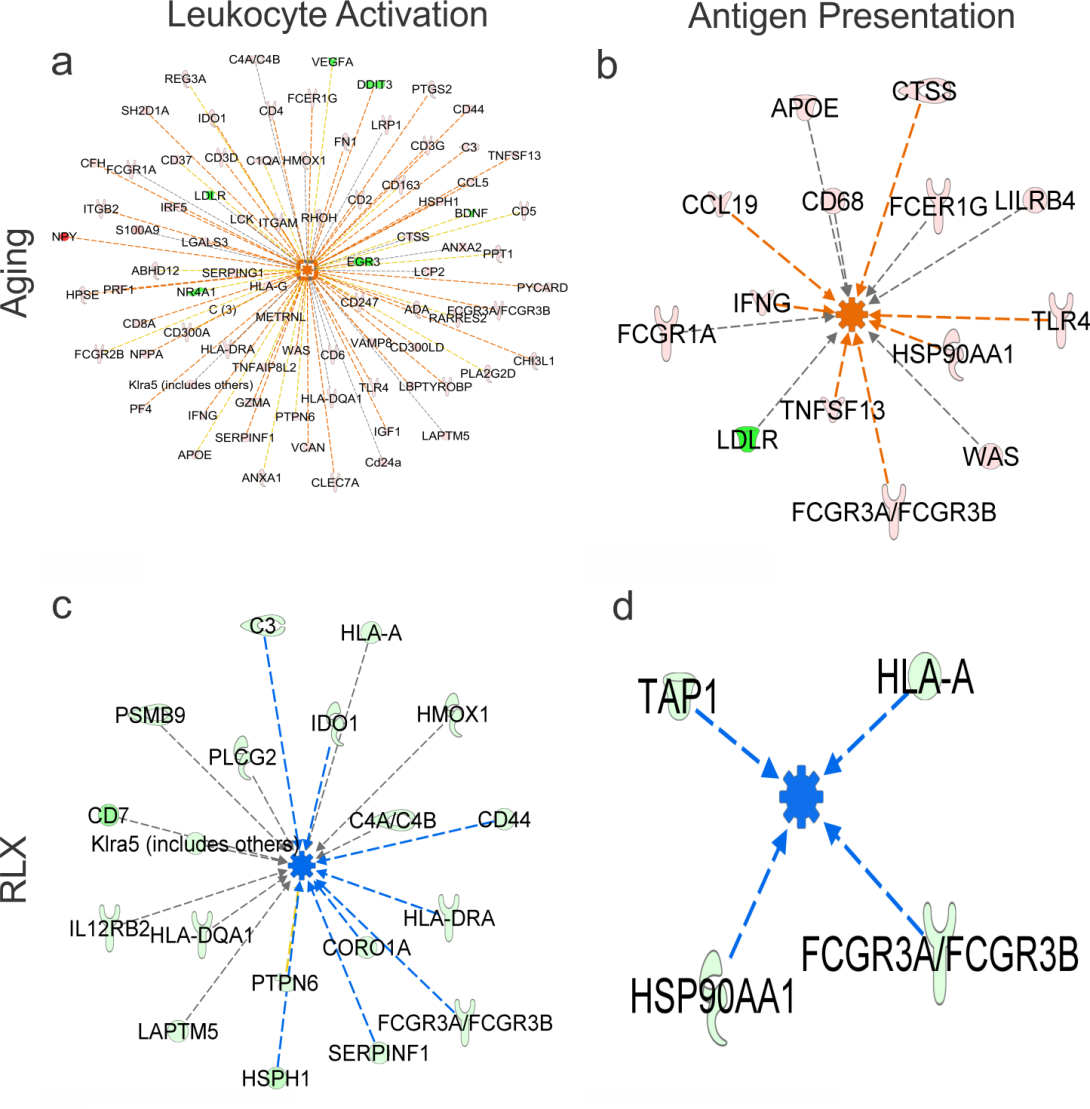
DEG analysis in female LV to predict cellular functions. IPA shows that aging leads to extensive alterations of gene expression resulting in leukocyte activation (panel a) and antigen presentation (panel b). RLX significantly reduced the expression of multiple genes associated with immune activation (panel c) and antigen presentation (panel d); that is, RLX largely reversed the effect of aging on the immune cell response. Orange centers denote activated function. Blue centers denote inhibited function. Green genes indicate reduced expression. Red genes indicated increases in expression. Orange, Blue and Yellow lines: the altered genes lead to activation, inhibition of function or is acting in a way opposite to the current literature, respectively. Brown lines: the function of the gene is unclear in the literature.

Analysis of DEGs by IPA to predict which cellular functions would be altered in aging or by RLX, showed that aged female rats exhibit increased gene expression involved in migration and activation of leukocytes and increased antigen presentation (Fig 4A and 4B), and these functions are significantly reversed by RLX (Fig 4C and 4D), supporting RLXs role in MHC regulation.

**Fig 5:**
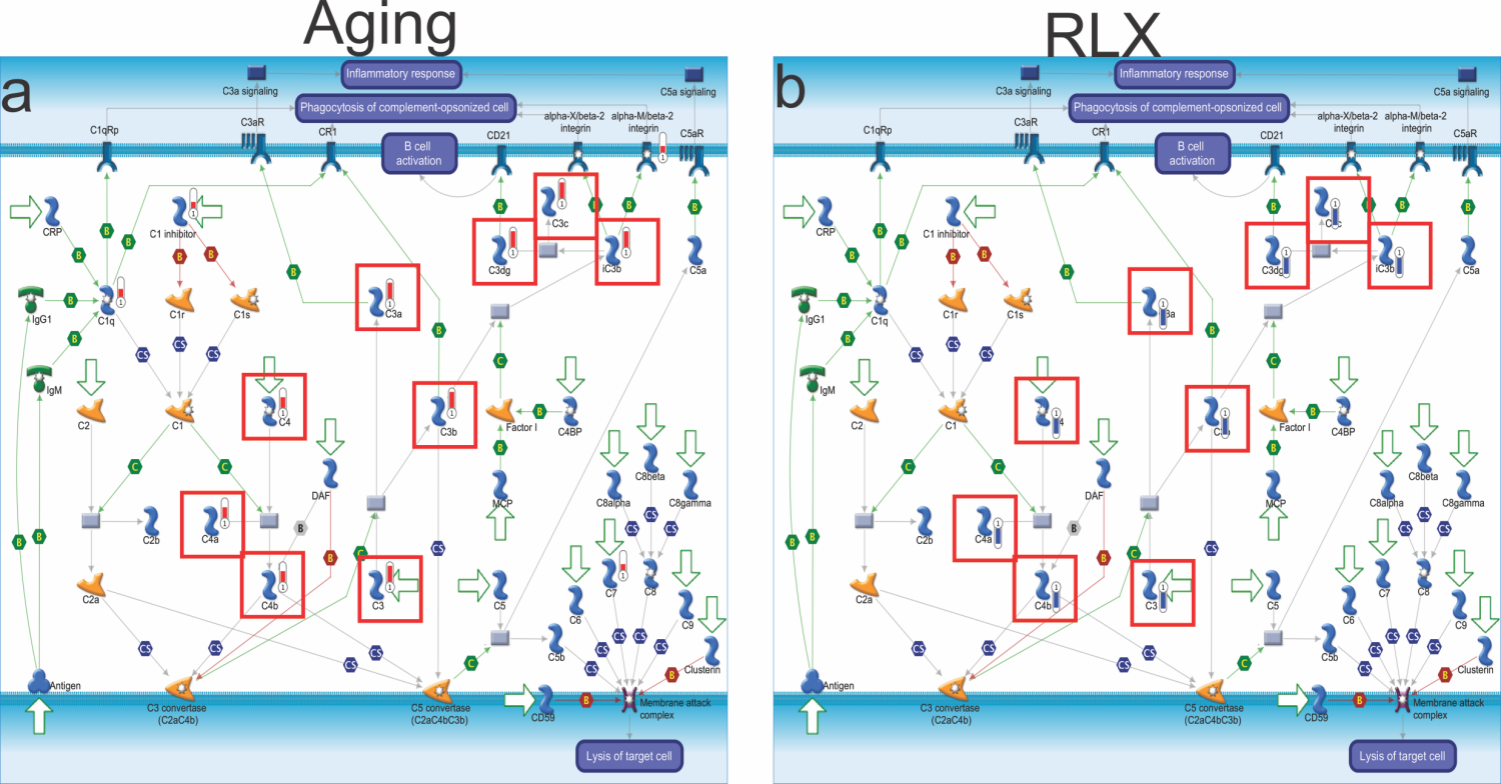
Classical complement pathway activity in female LV in aging and aging plus RLX. The classical complement cascade can result in an opsonization, or marking of cells for phagocytosis, and lysis of bacteria. It can also result in an augmented inflammatory response. A. Effect of aging on complement component activation. B. Effect of RLX on reversal of complement activity. Red boxes indicate significant alterations of specific complement components. Red and blue thermometer markers next to specific components indicate increased and decreased component expression, respectively. For key describing complement components, see https://portal.genego.com/help/MC_legend.pdf.

In addition to the signaling pathways that are reversed by RLX based on IPA, MetaCore analysis also showed that the classical complement cascade is significantly activated in aging (Fig 5A) and reversed by RLX treatment (Fig 5B) in female rats.

### Male rats

**Fig 6:**
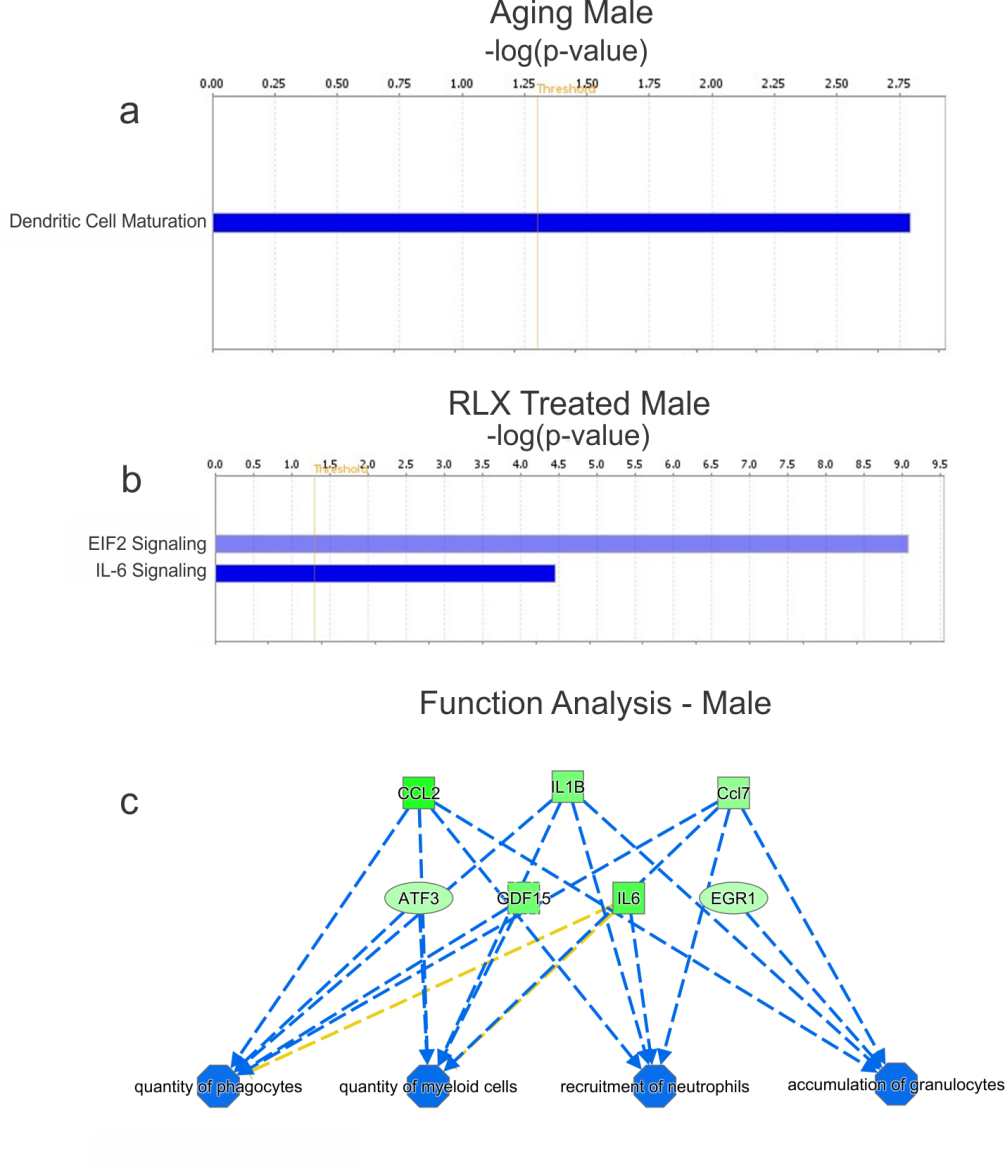
Relaxin suppress immune cell recruitment and accumulation, in male ventricles. Male rats had negligible pathway alterations (a) with aging or (b) RLX treatment. Blue bars indicate pathway inhibition. IPA in males (c) showed that quantity, recruitment and accumulation of various immune cells were inhibited (green) by RLX. In males, aging and RLX treatment altered 24 and 46 pathways, respectively; hence only pathways whose activation or inhibition was predicted by IPA (orange or blue colors, respectively) are shown here.

IPA pathway analysis of male rats (Fig 6A and 6B) show a significant decrease in dendritic cell maturation with age (Fig 6A) and inhibition of EIF2 and IL-6 signaling with RLX treatment (Fig 6B). These data, again, support the notion that there are major differences in the regulation of gene expression in male and female rats with aging and RLX treatment. MetaCore analysis in male rats suggest that multiple pathways are inhibited by RLX: IL-1, CCR1, CCL2, HMGB1/RAGE and LPS signaling, though these are not necessarily activated by aging (not shown). IPA analysis in male rats, did not show an activation of immune cell recruitment in aging, though RLX significantly repressed immune cell activity, recruitment and accumulation (Fig 6C).

These data and Tables 1–3 indicate that gender plays a vital role in terms of which genes are altered in aging and disease, and that treatments can affect males and females in different ways.

### Macrophage accumulation

**Fig 7:**
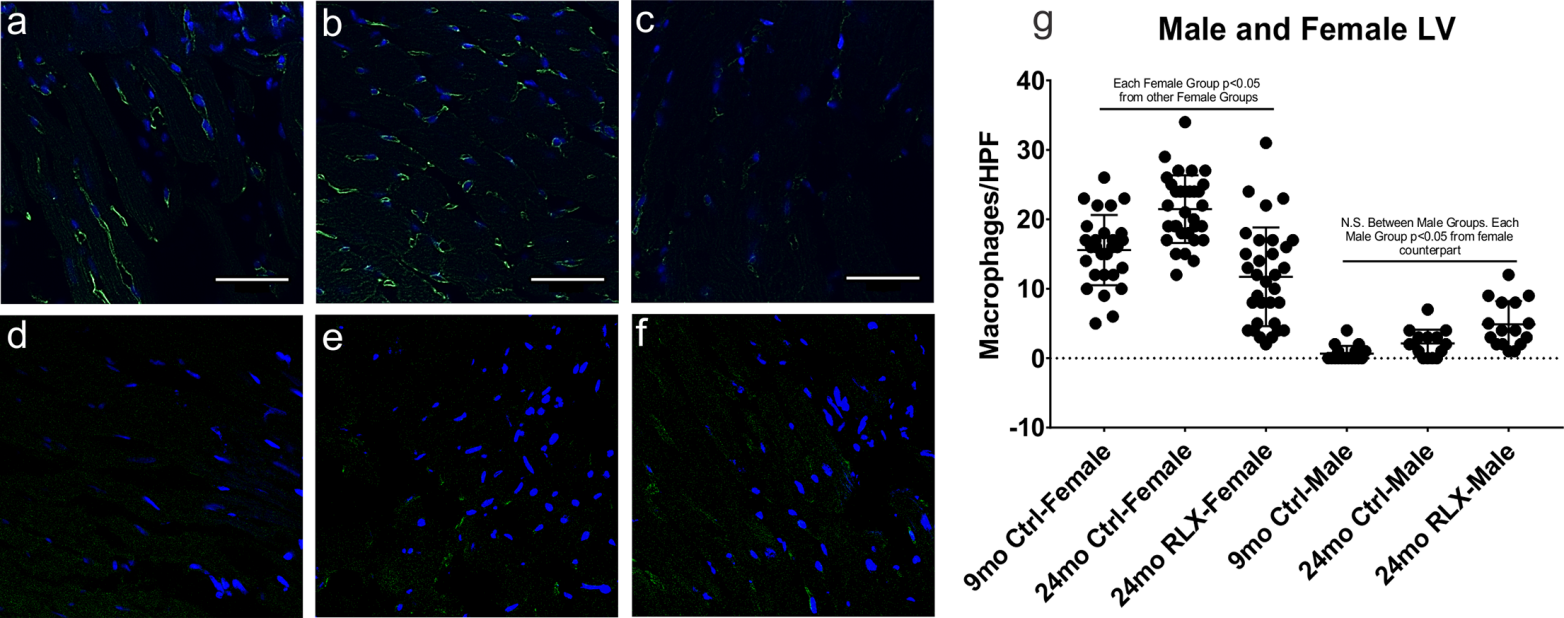
Effects of aging and RLX on macrophage accumulation in female ventricles. **A-C. Female.** Aged female left ventricles had significant macrophage accumulation (B. 21.47±0.86 macrophages/hpf) compared to young controls (A. 15.57±0.96) and RLX reversed this aged-dependent macrophage accumulation (C. 11.72±1.26). **D-F. Male.** Male left ventricles showed no change in macrophage accumulation in aging (E. 2.13±0.51) or with RLX treatment (4.88±0.84) compared to young (0.65±0.27) male rats, and had less macrophages than females in each group. G. Quantification of macrophage accumulation. Blue: DAPI; Green: F4/80 macrophage marker; HPF: high power field. Data is reported as mean ± SEM. N.S.: Not significant. 60x magnification. Scale bar: 50 μm.

Based on higher IFNγ, IL-6, MHC gene expression, increased complement activation, and prediction of increased inflammatory/immunce cell activity by IPA in aging *female* but not male rats, we first investigated the effects of aging on macrophage and T-cell recruitment in *female* ventricular tissue by immunofluorescence (Fig 7A–7C). Tissue macrophage infiltration measured with the marker F4/80 [50], increased significantly in aging (Fig 7B) compared to the young controls (Fig 7A) and was dramatically decreased with RLX treatment (Fig 7C). Next, we tested the male LV for macrophage accumulation (Fig 7D–7F). Male rats had significantly less macrophages that did the corresponding female rats in each group. Moreover, male aging (Fig 7E) did not significantly increase macrophage accumulation compared to the young male rats (Fig 7D), and RLX did not cause a significant change compared to young or aged male controls (Fig 7F). Although RLX slightly increased and decreased T-cell accumulation in females and males, respectively, the significance is unclear because T-cells were sparse (<6/high power field) in all groups (data not shown).

## Discussion

Our data show that aging promotes an inflammatory response in females, but not males, and RLX reverses these effects in females, and suppresses many inflammatory genes in *males*. One may reasonably conclude that RLX is acting at a genomic level to suppress inflammatory and immune responses in male and female rat heats. Others studies have bemoaned that only 10% of immunology studies consider sex as an important variable [51]; and the current findings re-enforce this concern by showing that females and males exhibit drastically different gene expression patterns and signaling pathway regulation, not only in aging, but also by relaxin therapy.

Reductions in IL-6 have been associated with beneficial effects in rheumatoid arthritis, though with increased LDL in the plasma.[13] Here, we showed that aging increased IL-6 transcription in females, while also significantly lowering the LDL receptor necessary to reduce LDL concentration in the plasma. However, RLX reduced IL-6 and increased LDLR, suggesting more LDL receptors and a greater efficacy at removing excess LDL cholesterol from the plasma. These results suggest RLX may prove to be a beneficial therapy for patients suffering from rheumatoid arthritis or coronary artery disease. In addition, IL-6 has been associated with type II diabetes development, and as such, RLX may prove to be a beneficial therapy in diabetes management [52].

The classical complement cascades converge on C3 components, which are necessary for C5 convertase formation and lead to inflammation (the anaphylatoxins C3a, C4a and C5a components) or bacteria lysis (C5b) [9]. Our data (Fig 5) shows that the C3a and C4a complement components are indeed two pieces, upregulated by aging and reversed by RLX. The anaphylatoxins can also increase activation of macrophages, resulting in increased release of inflammatory cytokines and stimulate the release of proteolytic enzymes which contribute to ongoing tissue damage and increase endothelial surface thrombogenicity. Moreover, clinical trials have shown that inhibition of C3 convertase attenuates both leukocyte attraction and mortality in cardiopulmonary bypass patients [9]. Taken together, our data suggest that relaxin could be a potent therapy to regulate aberrant complement activity.

Hofmann and Frantz [11] suggested that targeting specific cytokines in pre-clinical studies and clinical trials have failed to produce beneficial results due to the redundant nature of the immune system. Our study shows that RLX significantly alters multiple cytokines and signaling pathways in both genders, which could lead to improved suppression of inflammatory related pathophysiology that rely on these redundant immune effects.

Franceschi and Campisi [38] report that there are at least eight possible mechanisms for “inflammaging”: 1) persistent leukocyte production of reactive molecules, 2) cytokine production from damaged non-immune cells, 3) alterations in anabolic signaling, (e.g. TNFα and IL-6 signaling) altering insulin production, 4) “self-debris” from cell injury that accumulates over time, activating the inflammatory response, 5) reduced ability of the aged body to sequester infiltrated bacteria or viruses, 6) cellular senescence, 7) release of cytokines from damaged mitochondria and 8) dysregulation of the complement system. Our data supports reasons 1, 3 and 7 in that aging increases cytokine transcript production and RLX reduces cytokine transcript expression and accumulation of macrophages in LV tissue in females. Needless-to-say, further studies are needed on all possible mechanisms. Inflammation has been implicated in many aspects of cardiovascular disease including LV remodeling [53], hypertrophy [16], stroke [15], HF [14], and AF [54]. The suppression of inflammatory responses by RLX warrants further investigation considering its possible beneficial actions towards a therapy for such inflammatory related diseases as AF, diabetes, obesity and HF.

A limitation of this study is the lack of a general consensus for quantification and analysis of RNA-seq data [55]. The cut-off for filtering by RPKM is arbitrary and varies greatly in the literature, from 0.1 [56] to greater than 5 [57]. To determine which RPKM value to use for gene filtering, we obtained a freely available RNA-seq dataset analyzed using TopHat software [58], and compared results using our current analysis software. An RPKM ≥ 1 showed good agreement among the results between the two software packages. Another limitation is that RNA-seq was performed only on LV tissue, and it is unknown if atria or RV behave similarly. Finally, the experiments cannot discriminate whether RLX is removing the inflammatory stimulus resulting in downregulation of inflammatory markers, or is regulating transcription of the inflammatory markers themselves. The long-term effects following a short-term administration of RLX in clinical trials suggests a direct effect on transcription, and the broad reduction in multiple genes suggests a common nexus of action. Evidence suggests that multiple pro-inflammatory cytokines are controlled at the transcriptional level through histone modifications or phosphorylation.[59] Our lab showed that RLX can regulate phosphorylation of proteins [28], hence future studies should look into RLX’s ability to regulate histone modification and phosphorylation of genes.
